# Synchronous Papillary Thyroid Carcinoma and an Angiomatoid Fibrous Histiocytoma: A Rare, Interesting Case Report

**DOI:** 10.7759/cureus.112432

**Published:** 2026-07-10

**Authors:** Aliya Ishaq, Warda Siddiqi, Habibollah Aslami Nezhad, Iyad Hammadi, Arfan Al Awa

**Affiliations:** 1 Department of General Surgery, Canadian Specialist Hospital, Dubai, ARE; 2 College of Medicine, Mohammed Bin Rashid University of Medicine and Health Sciences, Dubai, ARE; 3 Department of General Surgery, Thumbay University Hospital, Graduate Medical Education, Ajman, ARE; 4 Department of Head and Neck and ENT Surgery, Dubai Hospital, Dubai, ARE; 5 Department of General Surgery, Dubai Hospital, Dubai, ARE

**Keywords:** angiomatid fibrous histiocytoma, case report, fibrohistiocytic tumor, neck mass, papillary thyroid carcinoma, surgical case reports, synchronous primary malignancies, thyroid nodule

## Abstract

Angiomatoid fibrous histiocytoma (AFH) is an uncommon fibrohistiocytic neoplasm of intermediate malignant potential that most often arises in the subcutaneous tissues of children and young adults. Involvement of the neck is unusual, and its clinical and radiological appearance may overlap with nodal or soft-tissue malignancy. Papillary thyroid carcinoma is the most common thyroid malignancy and commonly presents with suspicious thyroid nodules or cervical nodal disease. We report a 52-year-old man who presented with a gradually enlarging ulcerated left lateral neck mass and a small suspicious left thyroid nodule. Preoperative imaging showed a discrete thyroid lesion and a much larger necrotic lateral neck mass. Fine-needle aspiration of the thyroid nodule was suspicious for a follicular-patterned neoplasm, and the lateral mass was considered most likely to represent metastatic nodal disease. After multidisciplinary review, the patient underwent total thyroidectomy with excision of the lateral neck mass and neck dissection. Final histopathology demonstrated a 1.4-cm classic papillary thyroid carcinoma, staged pT1b pN0, with negative margins and no nodal metastasis. The lateral mass was a well-circumscribed fibrohistiocytic neoplasm with morphological and immunohistochemical features favoring AFH. The case represents the coincidence of having two different pathologies together when a patient presents with a lateral neck mass and thyroid malignancy. This emphasizes the need for an extensive workup for both pathologies separately, rather than taking a neck mass as a sequel of metastasis from thyroid malignancy.

AFH has a high incidence of local recurrence, and a small proportion presents with distal metastases; thus, close follow-up is required. At one year, the patient remained clinically well, with no evidence of neck recurrence, undetectable thyroglobulin, and a stable incidental pulmonary nodule. This case illustrates the importance of tissue diagnosis from anatomically distinct lesions when the size or appearance of a presumed metastatic deposit is disproportionate to the primary tumor.

## Introduction

Angiomatoid fibrous histiocytoma (AFH) is a rare soft-tissue neoplasm first described by Enzinger in 1979 as angiomatoid malignant fibrous histiocytoma [1]. It is now classified among tumors of uncertain differentiation with intermediate biological potential [2]. AFH typically presents as a slow-growing subcutaneous mass in the extremities, often in children or young adults [3,4]. Deep soft-tissue and head-and-neck presentations are uncommon, and because the lesion may contain hemorrhagic or cystic areas, it can mimic vascular tumors, sarcomas, or metastatic lymph nodes on imaging [3-5].

Papillary thyroid carcinoma (PTC) is the most frequent malignant thyroid tumor [6,7]. Cervical nodal metastasis is a familiar presentation in PTC, particularly when a lateral neck mass is identified with a suspicious thyroid nodule. However, a large lateral neck mass that is clearly disproportionate to a small intrathyroidal lesion should prompt consideration of a second pathology, especially when imaging features are atypical for nodal disease.

We describe a case of synchronous classic PTC and a large lateral neck fibrohistiocytic neoplasm with features favoring AFH. The case is clinically relevant because the lateral mass was initially managed as presumed metastatic disease, yet final pathology showed two distinct processes. The report is written to highlight diagnostic pitfalls rather than to imply a proven biological relationship between the two tumors.

As per the literature search, no definitive relationship between the two pathologies is reported, so it seems to be a coincidence in our case for both pathologies to be present at the same time.

This article was previously posted to the Research Square preprint server on January 28, 2026.

## Case presentation

A 52-year-old man presented with a slowly enlarging left anterior-lateral neck mass of approximately 12 months' duration. The mass had increased gradually in size and developed superficial ulceration with minimal discharge. He denied dysphagia, dyspnea, voice change, weight loss, previous neck irradiation, and family history of thyroid malignancy. He was clinically and biochemically euthyroid.

On examination, he was hemodynamically stable. Local neck examination showed a firm, non-tender, mobile mass measuring approximately 8 × 10 cm in the left lateral cervical region, with overlying skin ulceration. The mass did not move with deglutition or tongue protrusion. Because of its size, detailed palpation of the thyroid gland and cervical nodal levels was limited. No other cutaneous lesions or systemic abnormalities were identified. The gross clinical appearance of the lesion is shown in Figure 1.

**Figure 1:**
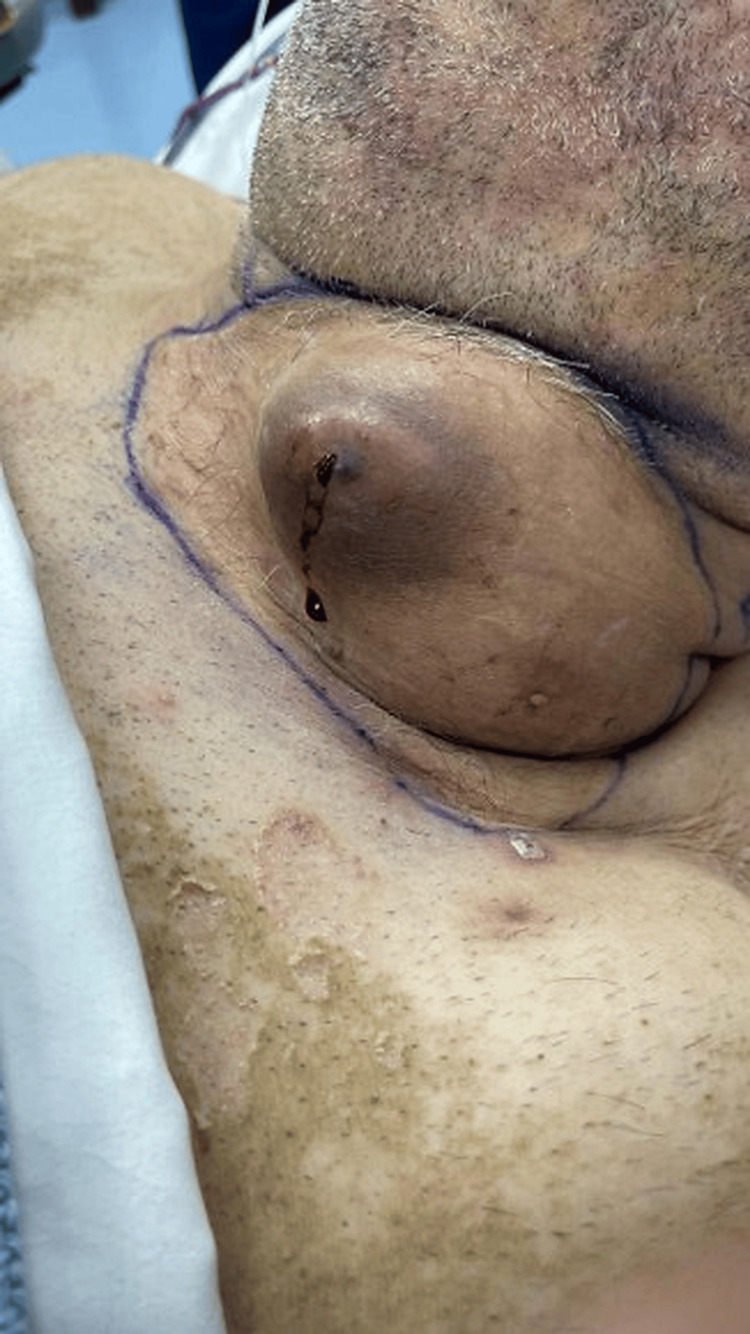
Gross clinical appearance of the left lateral neck mass showing superficial ulceration.

Diagnostic assessment

Neck ultrasonography demonstrated a solid hypoechoic left thyroid nodule in the upper posterior left lobe measuring 14.5 × 9.8 × 12 mm. The nodule contained echogenic and calcific foci and was categorized as Thyroid Imaging Reporting and Data System (TI-RADS) 4. A separate well-defined hypoechoic lateral neck lesion was also identified and appeared distinct from the usual jugular chain. Our radiology department follows the American College of Radiology (ACR) TI-RADS system for reporting thyroid nodules.

Fine-needle aspiration cytology of the thyroid nodule was reported as Bethesda IV, suspicious for a follicular-patterned neoplasm with papillary features. A contrast-enhanced computed tomography (CT) scan of the neck, chest, abdomen, and pelvis was performed to define the relationship of the lateral mass to adjacent structures and to assess for distant disease. CT confirmed a discrete thyroid nodule and a large adjacent soft-tissue mass measuring approximately 12 × 15 cm, with heterogeneous enhancement and central necrosis. The mass abutted adjacent vascular structures but did not show clear invasion. Representative coronal and axial CT images of the neck are shown in Figure 2 and Figure 3. No definite distant metastatic disease was identified. A small peripheral right-sided pulmonary nodule was noted and was considered indeterminate (Figure 4).

**Figure 2:**
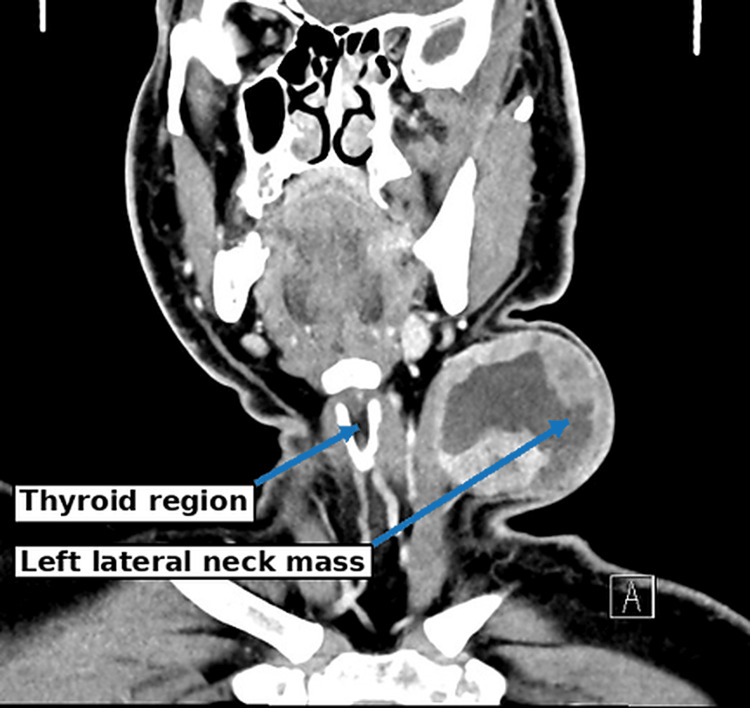
Coronal contrast-enhanced CT image showing a large heterogeneous left cervical mass lateral to the thyroid gland.

**Figure 3:**
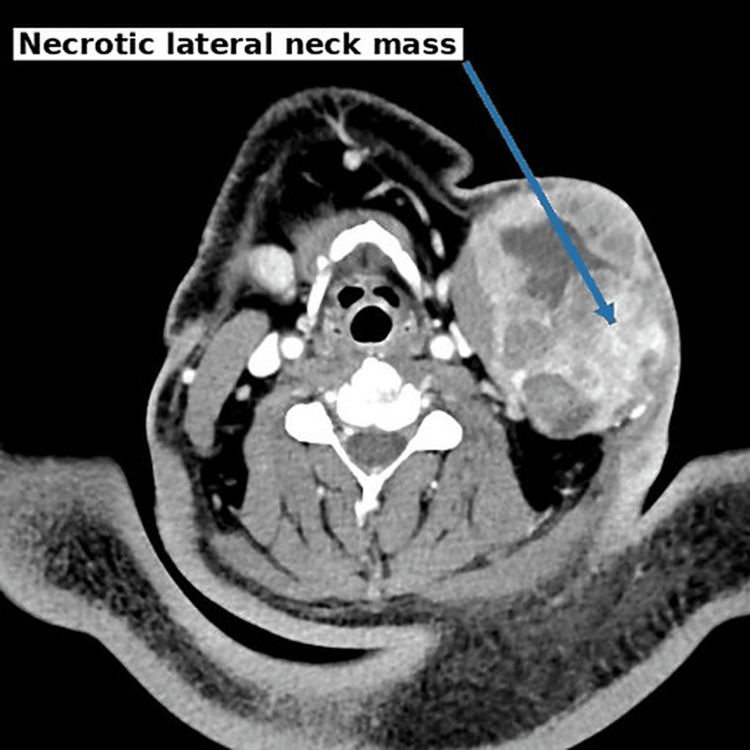
Axial contrast-enhanced CT image showing the necrotic heterogeneous left lateral neck mass.

**Figure 4:**
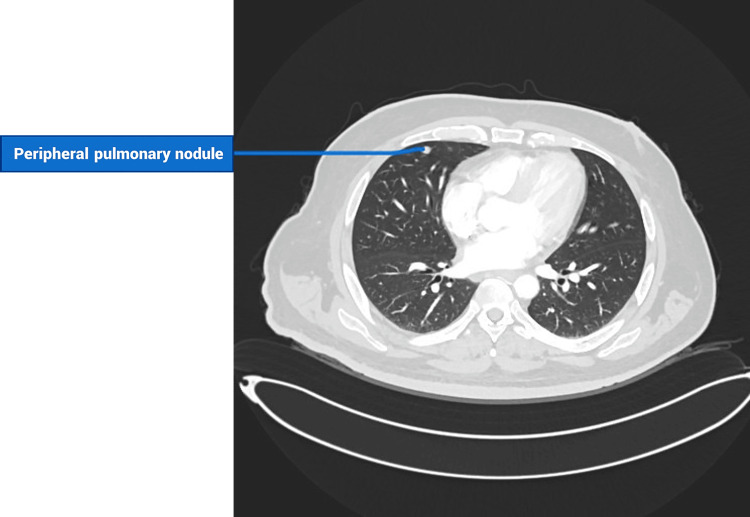
Axial chest CT image showing a small peripheral pulmonary nodule that remained stable on follow-up imaging.

Preoperative laryngoscopy showed bilateral mobile vocal folds, with no laryngeal lesion, deviation, or compression.

Therapeutic intervention

The case was discussed in a multidisciplinary meeting involving general surgery, head-and-neck surgery, endocrinology, radiology, and oncology. Metastatic PTC to cervical lymph nodes was considered the most likely diagnosis, although the possibility of a separate lateral neck pathology was also raised. Because the lesion was large, symptomatic, and closely related to important neck structures, the consensus plan was total thyroidectomy with excision of the lateral neck mass and neck dissection rather than additional preoperative biopsy of the lateral mass.

Intraoperatively, the thyroid lesion was confined to the left thyroid lobe without obvious extrathyroidal extension. The lateral lesion was firm, encapsulated, and separate from the lymphatic chains. These findings raised suspicion of a mesenchymal lesion rather than nodal metastasis. Representative intraoperative views of the mobilized mass and the operative bed after excision are shown in Figure 5 and Figure 6. The recurrent laryngeal nerves and parathyroid glands were identified and preserved.

**Figure 5:**
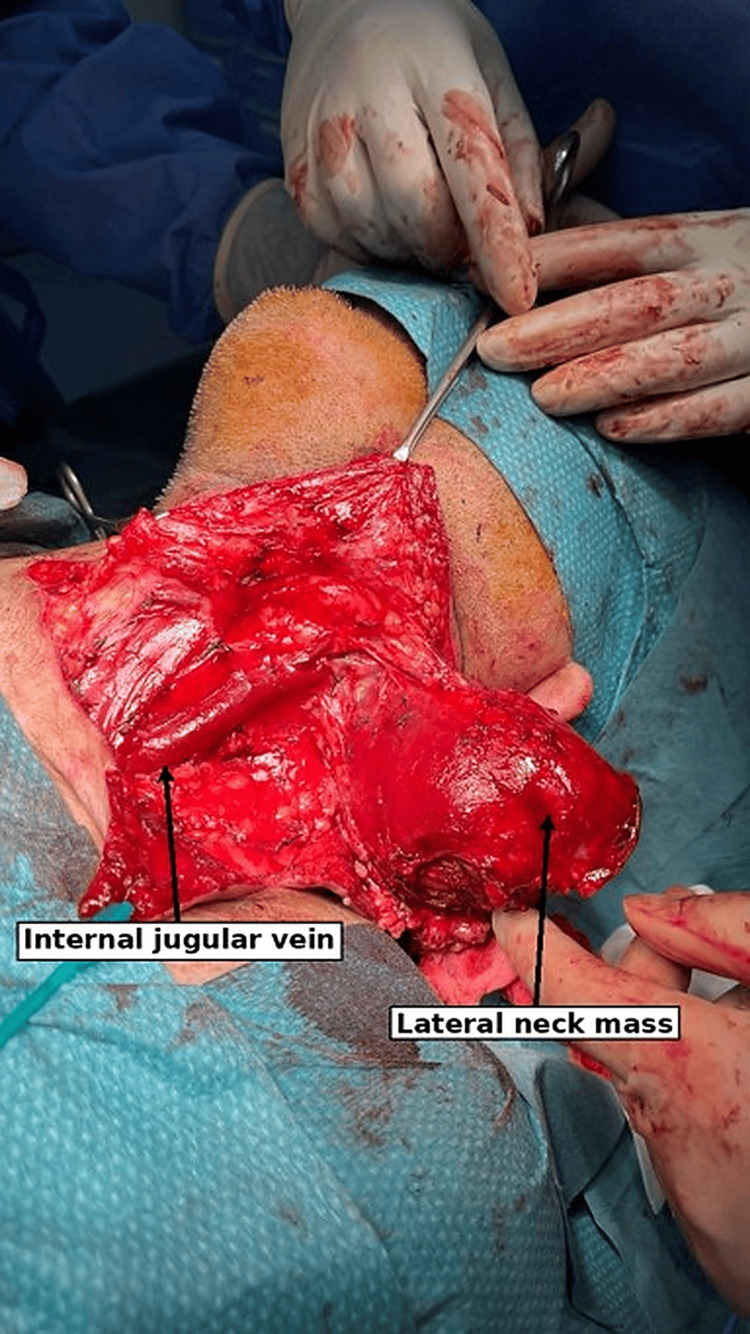
Intraoperative view demonstrating the mobilized lateral neck mass and its relationship to the internal jugular vein.

**Figure 6:**
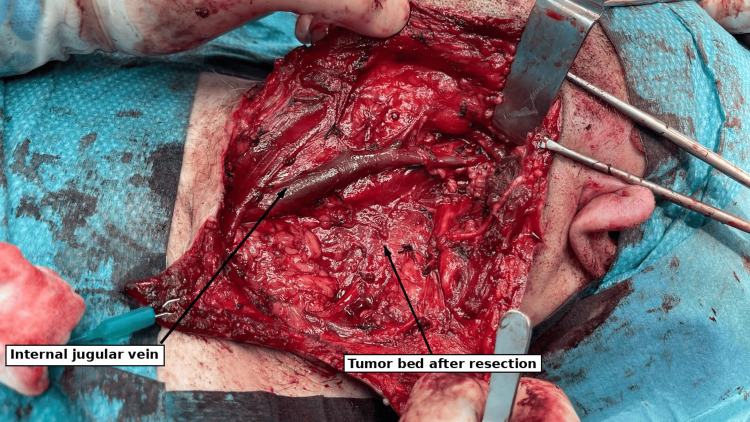
Operative bed after excision of the left lateral neck mass.

Pathological findings

Histopathological examination of the thyroid showed classic PTC, unifocal, located in the left lobe and measuring 1.4 cm in greatest dimension. The gross thyroidectomy specimen is shown in Figure 7. There was no lymphovascular invasion, perineural invasion, or extrathyroidal extension, and the surgical margins were free of carcinoma. Twelve regional lymph nodes were examined and were negative for metastasis. The thyroid carcinoma was staged as pT1b pN0m0 according to the eighth edition of the American Joint Committee on Cancer staging system [8].

**Figure 7:**
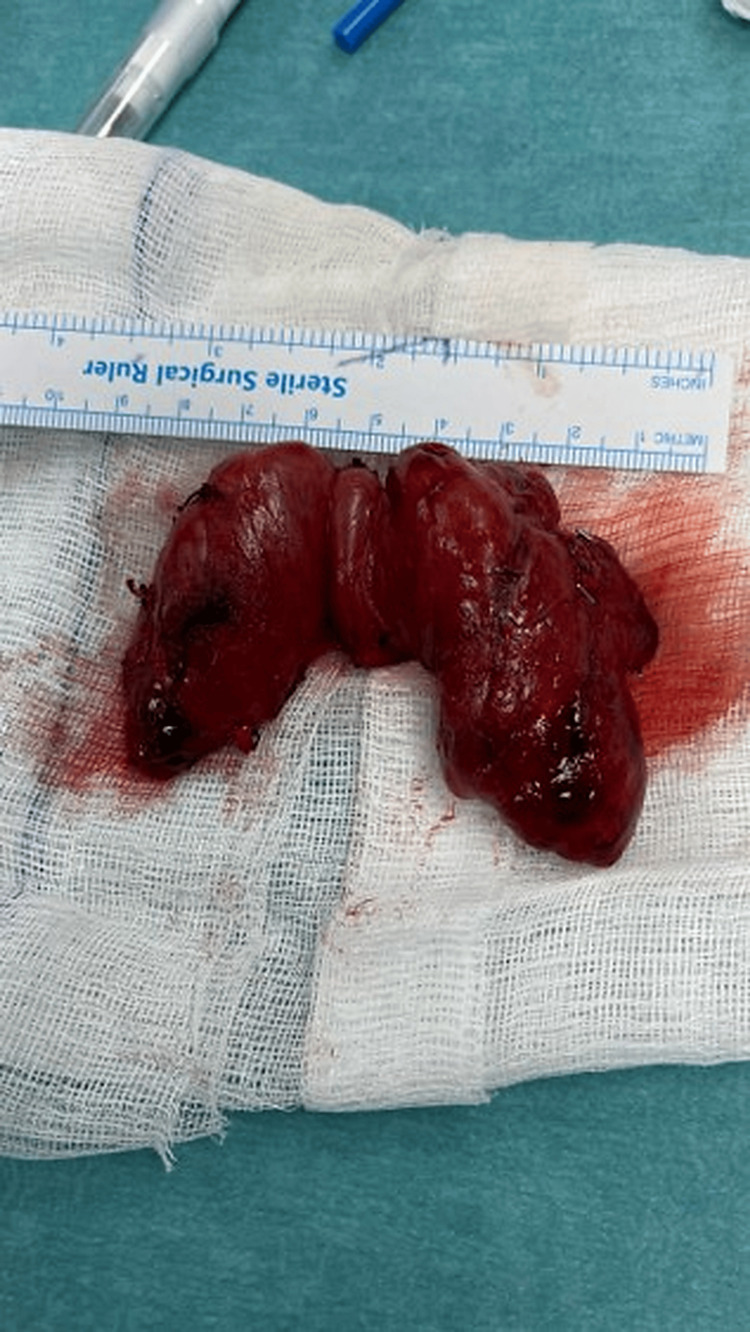
Gross thyroidectomy specimen. The small intrathyroidal lesion was separate from the larger lateral neck mass.

The left lateral neck mass measured 10 cm and was described as a well-defined, encapsulated fibrohistiocytic neoplasm based in the subcutaneous tissue, with expansile dermal and deep soft-tissue components. The lesion showed extensive secondary degenerative and hemorrhage-related changes, a foreign-body giant-cell reaction, xanthogranulomatous and fibroinflammatory changes, chronic peripheral inflammation, hemosiderosis, fibrosis, and central cystic change containing cholesterol clefts. Skin, fat, and skeletal muscle were not involved, and the resection margins were free of tumor. Lymph nodes sampled from the left neck, left supraclavicular region, right level IV, and right level II showed reactive hyperplasia only and no metastasis.

Immunohistochemistry was performed with cytokeratin AE1/AE3, S-100 protein, Melan-A, factor XIIIa, CD34, INI1, ALK, HHV8, CD68, smooth muscle actin, CD31, desmin, and ERG. The lesion showed strong diffuse desmin expression, with positivity for factor XIIIa and CD68. CD31 and CD34 highlighted vascular components rather than supporting an epithelial or thyroid origin. The remaining markers were negative. The integrated diagnosis favored AFH over atypical fibroxanthoma and undifferentiated pleomorphic sarcoma. Molecular testing for EWSR1 rearrangement was not performed.

Follow-up and outcomes

The postoperative recovery was uneventful. The patient had no vocal cord palsy or clinical hypocalcemia and was discharged on postoperative day three on levothyroxine suppression therapy. At six months, he remained asymptomatic. Neck ultrasonography showed no residual or recurrent disease in the thyroid bed or cervical compartments, and serum thyroglobulin was undetectable. At one year, clinical follow-up remained unremarkable. The previously noted pulmonary nodule was unchanged on repeat chest CT and was considered insignificant after multidisciplinary review. 

Since fibrohistiocytomas have a tendency for local recurrence and a small proportion can also metastasize, the patient is asked to undergo follow-up every three months and to repeat a neck CT after one year or earlier if needed. Further investigation for metastasis will be performed if indicated on clinical or radiological grounds.

## Discussion

This case presented an unusual diagnostic problem: a small suspicious thyroid nodule coexisted with a much larger lateral neck mass. In routine thyroid cancer practice, a lateral cystic or necrotic neck mass may be interpreted as nodal metastasis, particularly when a suspicious thyroid lesion is present. In this patient, however, the size, superficial involvement, and separation of the mass from expected lymphatic chains were features that later proved important. The final pathology demonstrated a small node-negative PTC and a separate fibrohistiocytic neoplasm favoring AFH.

AFH is uncommon and may be under-recognized outside its typical subcutaneous extremity location [3,4]. Classical histological features include a multinodular or syncytial spindle-cell proliferation, blood-filled pseudoangiomatous spaces, a fibrous pseudocapsule, hemosiderin deposition, and a lymphoplasmacytic cuff [3,4]. Not all features are present in every case, and degenerative or hemorrhagic change may complicate diagnosis. Immunohistochemistry is useful mainly to exclude mimics, including epithelial, melanocytic, vascular, myogenic, and high-grade pleomorphic tumors. Molecular confirmation with demonstration of EWSR1 rearrangement can support the diagnosis, but it was not available in this case [4,5]. For that reason, the diagnosis is best framed as a fibrohistiocytic neoplasm with features favoring AFH rather than as a molecularly confirmed AFH.

The main clinical lesson is not that PTC and AFH are biologically linked; there is insufficient evidence to make that claim. Rather, the case shows that synchronous pathology can occur in the same anatomical region and can distort preoperative staging. A large presumed nodal deposit that is disproportionate to a small primary thyroid lesion should prompt consideration of a second lesion. When technically feasible, separate tissue sampling from both the thyroid nodule and the lateral neck mass may prevent inaccurate staging and may help tailor the extent of surgery.

At the same time, the decision-making in this case should be interpreted in context. The lateral mass was large, ulcerated, symptomatic, and closely related to major cervical structures. Even if a preoperative biopsy had been attempted, core biopsy of AFH-like lesions may be non-diagnostic because of hemorrhage, cystic degeneration, and sampling limitations. The definitive diagnosis ultimately required complete excision and full histopathological assessment. The absence of nodal metastasis also altered the interpretation of the pulmonary nodule, which remained stable and clinically insignificant on follow-up. However, close observation and follow-up are advised for patients with repeat neck imaging after one year and further investigations if indicated on clinical and radiological grounds.

Current literature lacks any incidence of concomitant thyroid cancer and fibrihistiocytoma. Our case is probably a coincidence of having both pathologies together rather than any syndromic or genetic association, as it is not evident in the literature. However, it brings our attention to the fact that two pathologies can coexist, and in such cases, thorough radiological and pathological examination should be done after a multidisciplinary team meeting. When indicated, separate biopsies should be done for both thyroid mass and lateral neck mass, whether it is nodal metastasis from primary pathology or a separate nodal mass raising concern for a different pathology.

## Conclusions

This case describes synchronous classic PTC and a separate large lateral neck fibrohistiocytic neoplasm with features favoring AFH. The lateral mass clinically and radiologically mimicked nodal disease, but final pathology showed node-negative thyroid carcinoma and a distinct soft-tissue tumor.

The case supports a practical diagnostic principle: when a presumed metastatic deposit is unusually large, superficial, necrotic, or disproportionate to the primary thyroid lesion, clinicians should consider separate tissue diagnosis when feasible. Careful correlation of examination, imaging, operative findings, histopathology, immunohistochemistry, and molecular testing when available is essential to avoid misclassification and overtreatment. Rare mesenchymal tumors like AFH should be kept in the differential of atypical, cystic cervical masses, even in the setting of confirmed concurrent epithelial thyroid malignancies.
